# Nasal Osteotomies Revisited in Asians: Surface Aesthetics, Anatomical and Technical Considerations

**DOI:** 10.1055/a-2201-8219

**Published:** 2024-02-28

**Authors:** Jae-Yong Jeong, Taek-Kyun Kim, Inhoe Ku, Bakhtiyor Najmiddinov

**Affiliations:** 1 THE PLUS Plastic Surgery Clinic, Seoul, Republic of Korea; 2 Plastic and Reconstructive Surgery, Shox International Hospital, Tashkent, Uzbekistan

**Keywords:** rhinoplasty, osteotomy, nose

## Abstract

**Background** Although osteotomy is commonly performed in rhinoplasty, it is difficult for less experienced surgeon to understand mechanism of the procedure. The primary goal of this study is to improve understanding of nasal osteotomy in Asians by considering the surface aesthetics and anatomy of the nose as well as their relationships with the surgical procedure.

**Methods** Surface aesthetics, anatomic considerations, kinetics of medial and lateral osteotomy, fracture levels of osteotomy were discussed in detail by reviewing the previous publications and 18 years of our experience. Moreover, the technical details of osteotomy were explained and personal tips for performing successful osteotomy were described.

**Results** Dorsal and lateral aesthetic lines, dorsal and basal widths are main characteristics related to the surface aesthetics of nose to perform the osteotomy. In addition, these features are different in Asian population due to the anatomic difference with Caucasians, which makes the procedure difficult and requires more attention to perform osteotomy.

**Conclusion** Because osteotomy is one of the most traumatic and invasive part of the rhinoplasty, it is crucial for the rhinoplasty surgeon to understand the relationship between surface aesthetics and osteotomy techniques to produce consistent and reproducible results.

As the entire rhinoplasty procedure is similar to that of architecture and sculpturing, an osteotomy is one of the crucial parts of nasal sculpturing, which is the most traumatic and least controllable procedure in rhinoplasty. 1 Osteotomy is the procedure for adjusting the internal bony structure to change its reflection on the external appearance. In other words, the surgical goal is to ensure appropriate mobilization of the osteotomized bone without any irregularities or instability. The ideal osteotomy should incorporate successful bony mobilization as well as the stability of osteotomized bone. Generally, indications for nasal osteotomies include narrowing a wide dorsum, sealing an open-roof deformity after dorsal hump reduction, and correcting deviation of the bony nasal vault that is significant for both aesthetic and functional purposes. 2 3

Since it is difficult to control the slope of dorsal contour with osteotomy, thus results may be unfavorable, or unwanted problems may occur if osteotomy technique is not familiar. Even though several techniques of osteotomy have been described, it is hardly understood by the beginners because references describing the details of osteotomy skills are not enough. In addition, nasal bones are relatively short in length and low in height among Asians, and overlapped portion of keystone area is shorter than Caucasians, 4 5 which necessitates the delicate and precise osteotomy performance in Asian rhinoplasty.

The primary goal of this article is to improve the understanding about nasal osteotomy in Asians by considering the surface aesthetics and anatomy of the nose as well as their relationships with the surgical procedure. Therefore, we describe nasal osteotomies focusing on the technical as well as kinetic considerations based on 18 years of experience along with revisiting our previous studies. 2 3

## Surface Aesthetics for Nasal Osteotomies

### Dorsal Aesthetic Line and Lateral Aesthetic Line

**Fig. 1:**
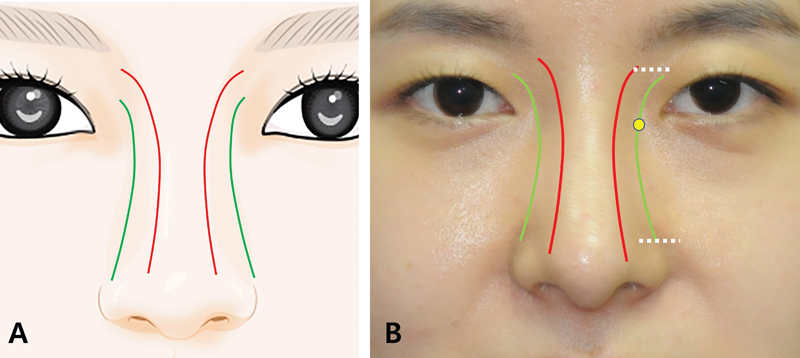
Dorsal aesthetic line (DAL) and lateral aesthetic line (LAL). ( **A** ) Graphical illustration of DAL and LAL. DALs start from supraorbital ridges superiorly and run medially along the glabellar area, eventually meeting at the level of medial canthal ligaments before subsequently diverging at the keystone area and finally, end up at the tip-defining points. The LAL lies on the nasofacial groove and demonstrates the transition line between the facial surface and lateral nasal polygons in surface aesthetics. ( **B** ) Topography of DAL and LAL on patient's nose. Vertically, the LAL starts from supratarsal sulcus (upper white dotted line) and after passing the medial commissure, it lies on the nasofacial groove. Caudally, the LAL extends down to the level of the supra-alar groove (lower white dotted line). Red line represents DAL, green line represents LAL, and the yellow dot indicates the location of the medial commissure.

As defined by Rohrich et al, the dorsal aesthetic lines (DALs) originate on the supraorbital ridges and run medially along the glabellar area, eventually meeting at the level of medial canthal ligaments before subsequently diverging at the keystone area and then, end up at the tip-defining points 6 ( Fig. 1A, B ). Sheen and Sheen described the DALs as “two divergent concave lines that are unbroken extensions of the superciliary ridges, which connect the radix with the lateral projection of the crura.” 7 The topography of the frontal bone, nasal bones, and upper lateral cartilages are the main defining factors of the nasal DAL. 8 In comparison to women, the DAL in men is wider and straighter, with less concavity at the superciliary ridges. 9 Asian noses have a wider and less straight DAL, with more concavity at the supraciliary ridges. 10 DAL can be changed according to variable procedures such as dorsal or lateral hump reduction, radix or dorsal onlay grafts, osteotomies, and filler injection.

In the meantime, aesthetics of the lateral nasal wall have not been clearly defined yet. Although Çakir et al described the nasal polygons and mentioned about the lateral aesthetic lines (LALs), the exact description about this LAL has not been explained. 11 According to Gerbault et al, the LAL shows the nasofacial groove, which connects the angulated frontal process of the maxilla to the maxilla itself. 12 The lateral bony wall, which encompasses the frontal process of the maxilla and the lateral portion of the nasal bones, with its associated shadowing, connects the DAL and LAL. Therefore, we have defined the LAL as a line, which lies on the nasofacial groove and demonstrates the transition line between the facial surface and lateral nasal (lateral bony and lateral cartilaginous) polygons in surface aesthetics (not bony landmark). Vertically, the LAL starts from supratarsal sulcus and after passing the medial commissure, it lies on the nasofacial groove. Caudally, the LAL extends down to the level of the supra-alar groove ( Fig. 1A, B ).

### Dorsal Width and Basal Width

**Fig. 2:**
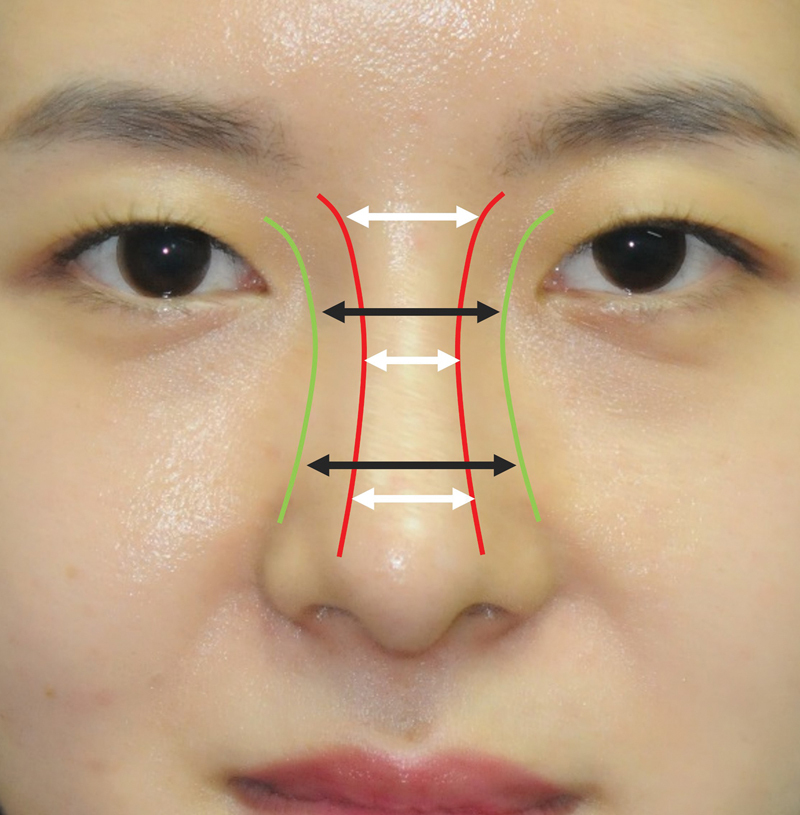
Illustration of dorsal width (DW) and basal width (BW). White arrow represents DW, while the black arrow represents BW. The red line represents DAL, and the green line represents LAL. DAL, dorsal aesthetic line; LAL, lateral aesthetic line.

Other important features of the nose are dorsal width (DW) and basal width (BW). The DW is the distance between the bilateral DALs. Rohrich et al stated that the ideal width of the DALs should match the width of the tip-defining points or interphiltral distance. 6 The width of nasal root is two-thirds of the alar base width or two-thirds of the intercanthal distance in Asians, according to Suhk et al. 10 However, the DW varies in different levels: it is widest proximally, narrowest in the middle and equal to the width of the tip-defining points distally ( Fig. 2 ). Although the BW is assumed as the width of the bony base, which should be 70 to 80% of the alar base or intercanthal distance, 7 10 it is not completely the same with the bony base because of various thickness of Skin Soft Tissue Envelope in Asian nose. The true BW can be explained as the distance between the bilateral LALs, and it is not equally oriented along the nose as the bilateral LALs are not parallel lines. 7 The BW of the nose can be divided into two major levels: proximal, the distance between the LALs at the canthal level and distal, the line connecting the LALs at the level of the pyriform aperture. Externally, the supra-alar groove which is the caudal end of the LALs shows the margin of pyriform aperture.

## Anatomical Considerations for Nasal Osteotomies

### Nasal Bone and Pyriform Aperture

The nasal bony framework, also known as the bony vault, is composed of the nasal bones and the frontal process of the maxilla. The paired nasal bones, which compose the superior border of the pyriform aperture, are attached superiorly to the nasal process of the frontal bone, laterally to the frontal process of the maxilla that forms the inferior and lateral boarders of the pyriform aperture, and inferiorly to the pyriform aperture. 4 13 The medial border of the pyriform aperture is made by the rounded edge of the premaxilla bone and the sharper edge of the maxilla. Moreover, the fusion of these two bones forms the anterior nasal spine. 13

**Fig. 3:**
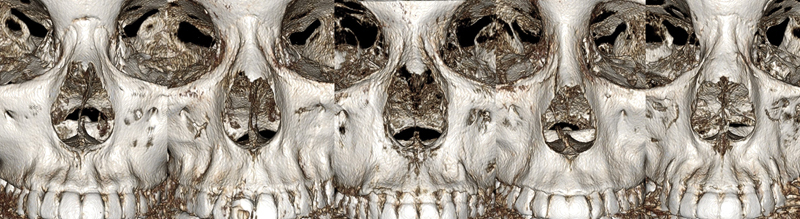
Various features of nasal bone and pyriform aperture in different individuals on 3D CT scan.

The three-dimensional configuration of the lateral bony wall should be considered when planning bony narrowing with osteotomies. Since the configuration of the lateral bony wall is different in each nose and even bilateral sides differ from each other in one nose with the frequent occurrence of the more prominent convexity on one side. 12 The reason is that the size and shape of the nasal bone and pyriform aperture differs depending on gender, race, and environmental factors 4 ( Fig. 3 ).

### Nasomaxillary Transition Zone

**Fig. 4:**
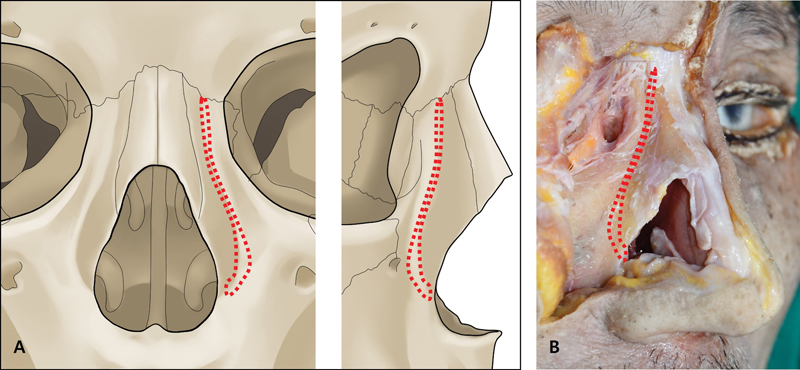
Nasomaxillary transition zone (NMTZ). ( **A** ) Illustration of NMTZ (red dotted line). ( **B** ) Demonstration of NMTZ in cadaver (red dotted line).

**Fig. 5:**
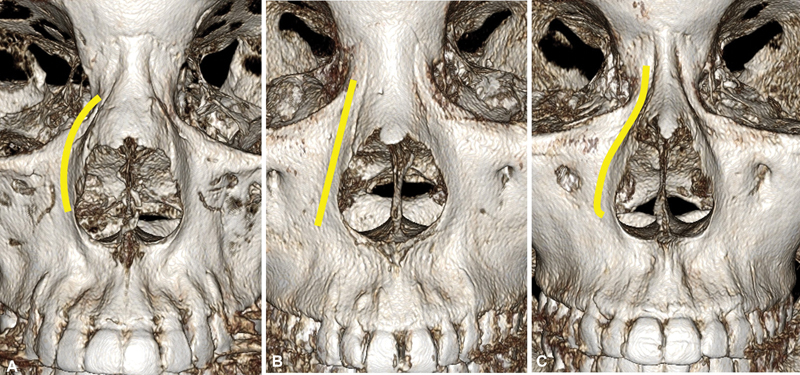
The configuration of the nasomaxillary transition zone can be described as ( **A** ) convex, ( **B** ) straight, or ( **C** ) concave–convex.

The frontal process of the maxilla forms a unique groove away from the nasomaxillary suture line as it goes down below the inferior orbital rim. In other words, there is an obvious transition area between the lateral nasal wall and anterior zygomatic surface. We named this groove as nasomaxillary transition zone (NMTZ) based on cadaver and CT study ( Fig. 4 ). This transition zone extends from medial canthal area to the margin of pyriform aperture. The lateral side of this area is a fossa-shaped depression, where the canine fossa is located, and it appears as a linear eminence and extends downward as a canine eminence. As the shape of this zone is determined by the configuration of the nasal and maxillary bones, the NMTZ can be shown as straight or curved line on CT images. In addition, the entire configuration of the NMTZ can be exposed as convex, straight, or concave–convex according to each patient ( Fig. 5 ). The clinical importance for rhinoplasty is that the transition zone is same as a safe zone for lateral osteotomy, which directly affects the LAL on the surface aesthetics. Therefore, it is recommended that all forms of lateral osteotomy should be performed within the NMTZ.

### Webster's Triangle

Controversy over whether bony medialization by osteotomy procedure affects airway function after surgery or not still exists among surgeons. In 1977, Webster et al suggested using a curved lateral osteotome that was positioned more superiorly, so he recommended to preserve a bony triangle at lower part of pyriform aperture to keep the airway open below the level of the inferior turbinates. 14 Gubisch, on the other hand, did not believe the efficacy of the bony triangle preservation to be a significant concern. 15 Gerbault et al also disagree about if this triangular portion must be preserved to maintain airway patency. 12

**Fig. 6:**
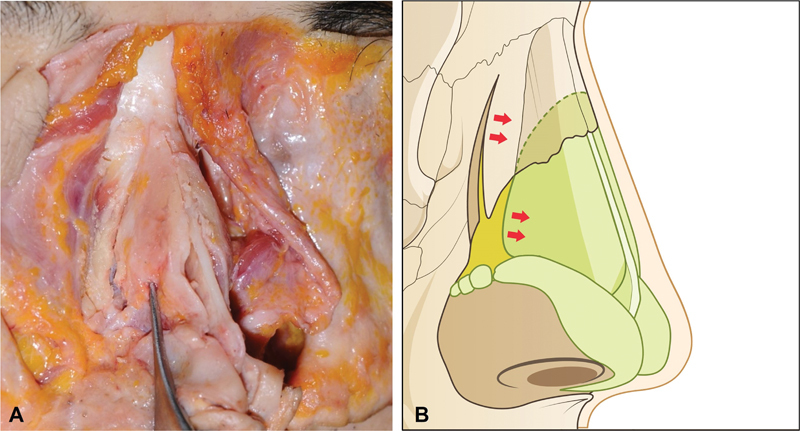
Relationship between osteotomy and upper lateral cartilage (ULC). ULC tends to move toward medial side with lateral osteotomy, because of overlapping between nasal bone and cartilage. It may cause potential narrowing of internal nasal valve due to hinge motion of osteotomized bone causing medial movement of ULC concomitantly. ( **A** ) Cadaver demonstration shows potential narrowing of internal nasal valve after osteotomy. ( **B** ) Illustration of the relationship between osteotomy and ULC.

The osteotomy procedure itself can potentially affect to airway compromise. First, as the amount of medialization of fractured bone increases, obstruction may occur, especially when performing medial and lateral osteotomy simultaneously. Second, after lateral osteotomies, the internal valve can be narrowed by inward movement of the upper lateral cartilage because of overlapping with nasal bone ( Fig. 6 ). Third, if the caudal starting level of the osteotomy at the pyriform aperture begins too low than the NMTZ, medialization of inferior turbinate can be accompanied.

**Fig. 7:**
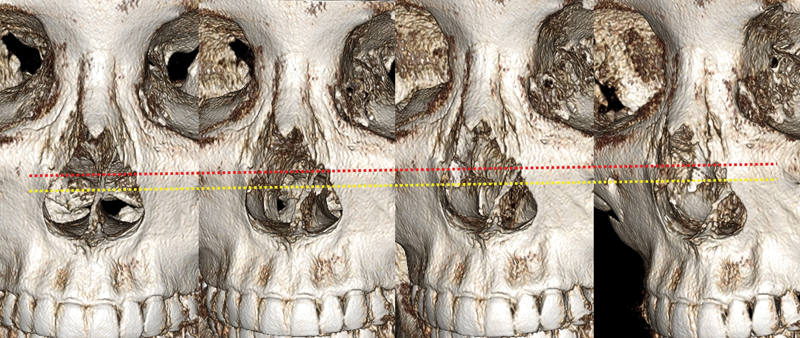
Serial photos showing the relationship between lateral osteotomy line (red dotted line) and attached level of inferior turbinate (yellow dotted line) after osteotomy.

However, under the senior author's experience, Asians encounter less airway problem than Caucasians after osteotomy though there is a debate with this issue. However, the wider angle of the internal valve in Asians may have an effect on it. 16 Therefore, we assume that the lateral osteotomy itself has minimal effect on airway obstruction ( Fig. 7 ). Among more than 3,000 cases who had osteotomies, we had few experiences related to postoperative airway problem. In addition, we usually have performed a preventive turbinoplasty using outfracture of the inferior turbinate neck and compression fracture of conchal bone to prevent airway problems after osteotomy, if there is any potential risk of airway obstruction after osteotomy procedure. 17

### Lacrimal Drainage System

**Fig. 8:**
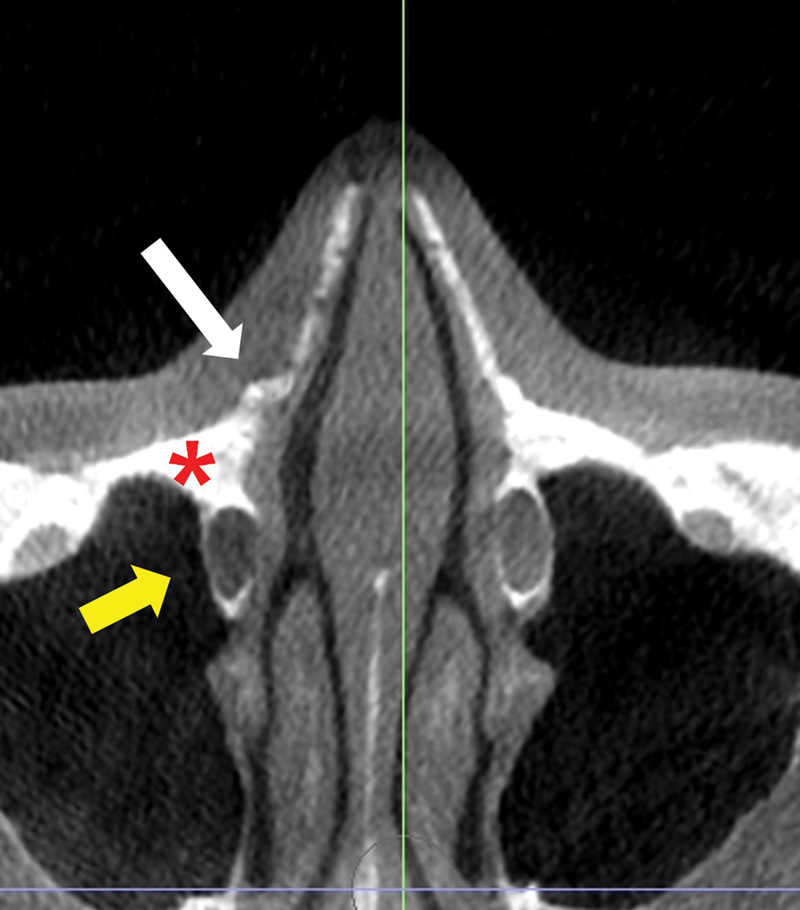
Relationship between osteotomy line and lacrimal apparatus. Lateral osteotomy can be performed within NMTZ since triangular buttress (red asterisk) protects lacrimal apparatus (yellow arrow). The white arrow indicates the fracture line over the NMTZ. NMTZ, nasomaxillary transition zone.

Another concern of performing osteotomy is the injury of the lacrimal drainage system. The lacrimal sac is located within the lacrimal groove, which is created by the lacrimal bone and the posterior edge of the frontal process of the maxilla. The nasolacrimal duct connects the palatine bone and inferior turbinate medially, and the maxillary bone laterally, to drain the lacrimal sac. The course of the nasolacrimal duct ends beneath the inferior turbinate, at the inferior meatus. 18 Since lacrimal crest is closely located and connected with medial portion of the inferior orbital rim, the chance of affect to the lacrimal sac or duct increases if low-to-low pattern osteotomy goes too much superiorly. However, unless the osteotomy line extends excessively deep laterally (below the NMTZ), iatrogenic injury to the lacrimal system is unusual during a lateral osteotomy. 19 In addition, the existence of a triangular buttress which is the thickening of maxillary bone protects the lacrimal apparatus. 20 Performing lateral osteotomy in this region is unacceptable as bony resistance is high and it may compromise the lacrimal ducts. As we explained ahead, the NMTZ is safe for the lacrimal system and it is easier to perform osteotomy in this region due to lower resistance ( Fig. 8 ).

The transient epiphora is caused by postoperative edema rather than damage to the lacrimal drainage system, and thus tends to resolve spontaneously. 20 According to report by Sachs, 2% of patients undergoing rhinoplasty experienced transient epiphora that resolved spontaneously within 6 months, while none of the patients experienced symptoms that lasted longer than 8 months after the operation. They also reported that lacrimal system injury can occur during subperiosteal tunneling on the lateral nasal wall or during lateral osteotomies with a saw. 21 Even though many patients suffered from temporary epiphora following nasal osteotomy during the past 18 years of experience, permanent epiphora or lacrimal system injury has never been observed. 2 3

## Relationship between Osteotomies and Surface Aesthetics

**Fig. 9:**
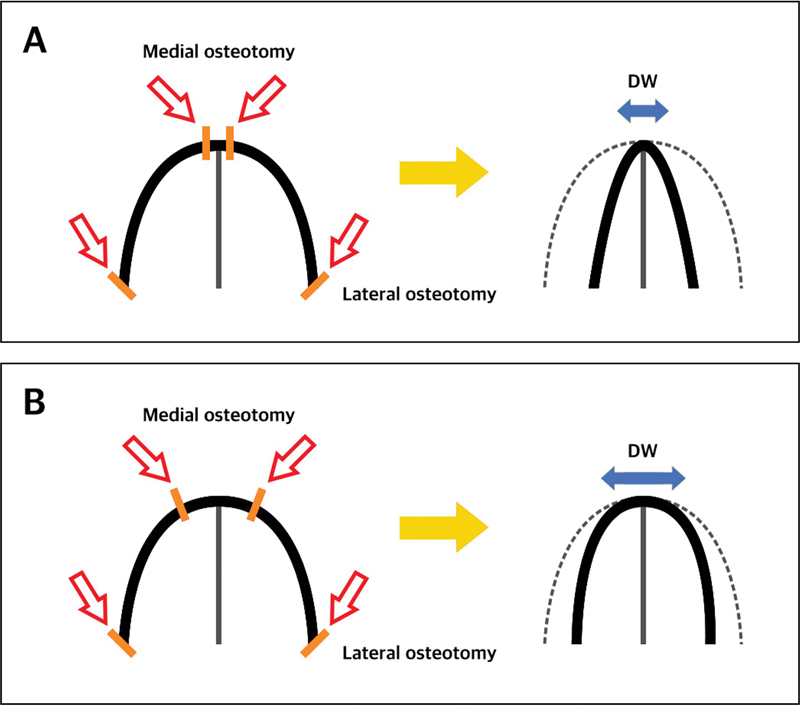
Difference of postoperative DW according to starting point of the medial osteotomy. (A) If the starting point of the medial osteotomy lines are closer to each other, resulting DW will be narrower. (B) If the starting point of the medial osteotomy lines are farther to each other, resulting DW will be wider. DW, dorsal width.

The ultimate goal of performing osteotomies is to change the surface aesthetics modifying DAL, LAL, DW, and BW. Therefore, surgeons need to understand how each osteotomy procedure directly affects surface aesthetics. When correcting the wide bony dorsum, lateral osteotomy alone directly affects the reduction of the BW, however it is minimally effective to reduce the DW or clarify the DAL. In other words, lateral osteotomy does not directly control the DAL but rather affects the LAL. If lateral osteotomy is performed together with medial osteotomy, combined osteotomies affect DAL and maximize the change of DW because the range of movement of osteotomized bone is increased by synergistic effect. Therefore, it is necessary to perform medial osteotomy as well if DW is wide. The starting point of the medial osteotomy plays an important role in adjusting the DW. For example, the closer the medial osteotomy line to each other, the narrower the DW after the osteotomy. In contrast, if bilateral medial osteotomies are applied far from each other, new DW will be wider ( Fig. 9 ).

**Fig. 10:**
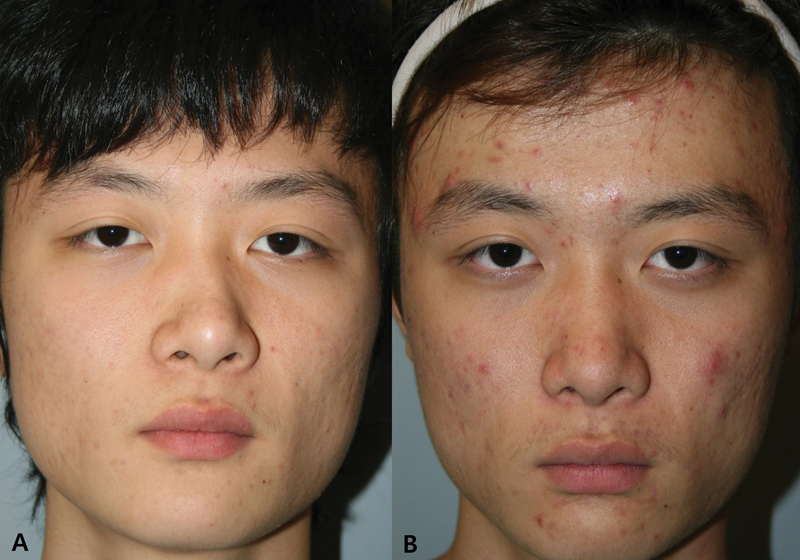
Changes in the dorsal aesthetic lines after deviated nose correction. ( **A** ) Preoperative view. ( **B** ) Postoperative view.

Medial osteotomy can be performed using an outfracture pattern to correct the alignment of deviated bone. This technique involves moving the overmedialized bone outward, resulting in a lateral shift of the DAL from the medial to the lateral direction ( Fig. 10 ). In addition, septoplasty should be performed concomitantly to obtain straight DAL in most of the cases.

**Fig. 11:**
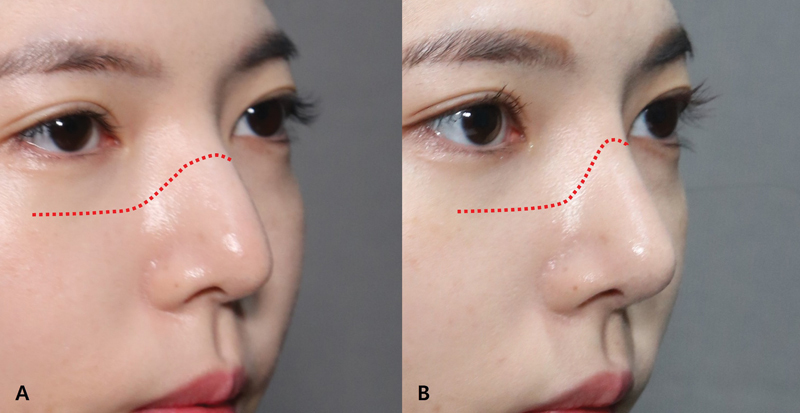
More deepening of the nasofacial groove was achieved by osteotomy, creating three-dimensional effect. Photos of a 26-year-old patient after osteotomies. ( **A** ) Preoperative view. ( **B** ) Postoperative view.

Changes in the LAL are associated with alterations in the inclination angle of the lateral nasal wall. These changes can be readily observed in frontal or oblique views both before and after surgery. It is shown that osteotomies can create three-dimensional effect by deepening of NMTZ ( Fig. 11 ).

## Technical Considerations for Nasal Osteotomies

### Basic Technical Considerations

**Fig. 12:**
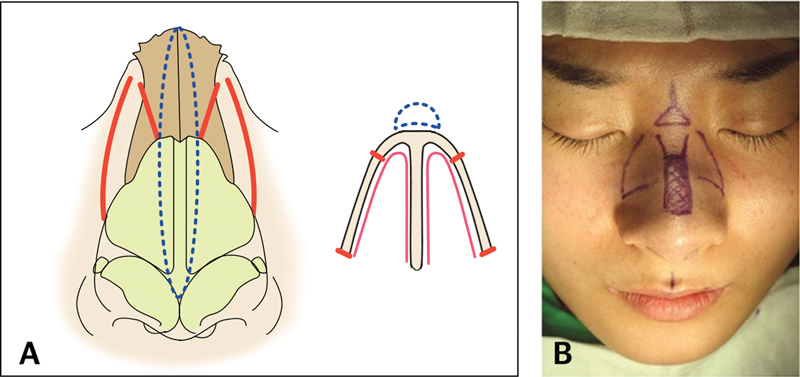
( **A** ) Illustration of authors' osteotomy technique including paramedian oblique osteotomy and low-to-low pattern lateral osteotomy. ( **B** ) Intraoperative design of osteotomies.

The medial osteotomy is a procedure to separate the nasal bones from midline bony septum. It is applied when the bony dorsum is deviated, excessively wide or narrow. For medial osteotomy, variable applications such as paramedian, medial, medial oblique, and transverse osteotomies can be used according to the purpose. We prefer paramedian oblique osteotomy and percutaneous lateral osteotomy for aesthetic nose 2 ( Fig. 12 ).

During medial osteotomy, if starting point of osteotomy begins too close to midline septum, they are more likely to disrupt the keystone area, causing weakness or damage it. On the other hand, we begin at 2 to 3 mm laterally from the midline as the paramedian oblique pattern, which is far enough away from the midline to keep the keystone structure stable and minimize injury to adjacent tissues. 22 23 In other words, this method provides stability by preserving keystone area, which enables to use the implant for dorsal augmentation with more stability in Asians. 2 3 Medial or medial oblique osteotomy is usually recommended to correct the bony septum when high septal deviation or severe bony deviation exists.

The lateral osteotomy can be performed through buccal, alar, vestibular, and percutaneous approaches. Two mostly performed techniques among lateral osteotomies are internal continuous osteotomy via vestibular approach and external osteotomy via percutaneous approach. During internal continuous osteotomy, the tip of the guarded osteotome initially sticks to the edge of the pyriform aperture, resulting in complete engagement of the burrow into the bone and complete fracture. When sufficient osteotomy is achieved, the osteotome is rotated inward to induce medial mobilization with a prying force. On the other hand, when performing percutaneous lateral osteotomy, a direct incision is made on the skin for engagement of the osteotome, and bony scratching for making grooved hatch is made using one corner of the sharp edge of the osteotome blade. Scratching is made by oblique stroke applied by hammering with mallet. 2 3

### Kinetics of Medial and Lateral Osteotomy



**Video 1** Demonstration of osteotomies in a skull model.


Lateral osteotomy modifies the LAL by modifying the previously described NMTZ. This zone is not located on the nasal bone, but the frontal process of the maxilla. It is not only the transition between the nose and maxilla, but also the transition zone of bony thickness along the lateral nasal wall. Anatomic studies demonstrated that thickness is less than 2.5 mm in this area and can be fractured using small osteotomes. 24 For optimizing the outcomes, lateral osteotomy should be performed as an ascending cutting line along the frontal process of the maxilla from the margin of pyriform aperture.

Medialization of the osteotomized bone can be maximized by separating the nasal bone from the bony septum using medial osteotomy first and then moving it with lateral osteotomy. When performing the medial osteotomy, it should be done prior to lateral osteotomy to maximize the mobilization of osteotomized bone toward medial side.

**Fig. 13:**
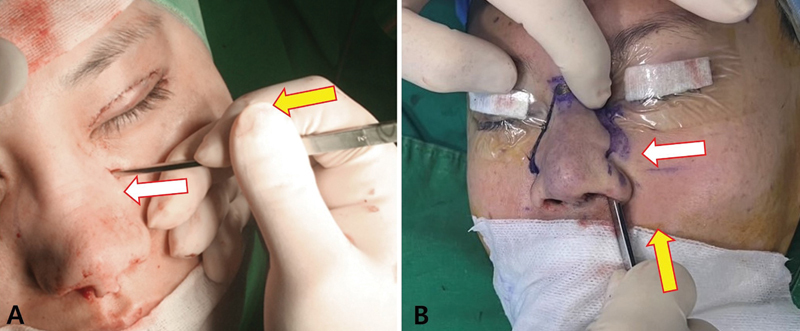
Different vector of each lateral osteotomies. ( **A** ) External lateral osteotomy. ( **B** ) Internal lateral osteotomy. Yellow arrow indicates the vector of power, while the white arrow represents the direction of movement of the fractured bone.

The kinetics of the osteotomized bone differs in external and internal lateral osteotomy. According to the type of osteotomy, vector of striking force is different ( Fig. 13 ). The vector of force given by mallet is not in a same direction with the vector of movement of the bone in internal continuous osteotomy: First, an osteotome is moved cephalad by hammering, and then, surgeon moves it inward to medialize the osteotomized bone. On the other hand, unidirectional vector of force and bony movement is achieved in percutaneous lateral osteotomy as the osteotome movement by hammering and bony movement are in the same direction which are toward the midline. Considering the direction of nasal bone movement in external lateral osteotomy, a hinge motion from the proximal pivot is ideal for movement of osteotomized bone ( Fig. 6 ). The additional medial or transverse osteotomy can maximize the medial movement of the hinge arch.

Many previous articles introduced fracture mechanism about percutaneous lateral osteotomy as the dotted pattern, which is not continuous. 23 However, we have experienced that fracture pattern by making a continuous grooved hatch is more advantageous in Asian nose than a dotted pattern. In other words, instead of being fractured in a perforating pattern, the fractured bone is moved through the steps of scratching, fracturing, and pushing. With repeated scratching at the same line with backward and forward motion, the osteotome is raised more vertically and fracture is induced using striking power in a weakened state. A surgeon can notice the bony movement by feeling transmitted through the osteotome while striking with mallet, and by listening a characteristic fracture sound ( Video 1 . Demonstration of osteotomies in a skull model). Finally, a fractured bone can be modified precisely with gentle finger pressure.

### Differences According to the Fracture Levels

**Fig. 14:**
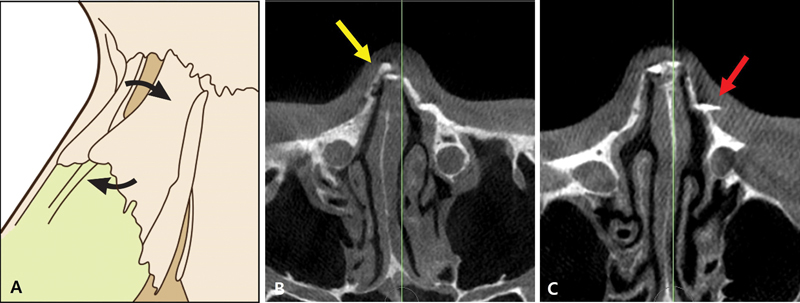
Rocker deformity and staircase deformity. ( **A** ) Illustration of rocker deformity and staircase deformity. ( **B** ) Rocker deformity on CT scan (yellow arrow). ( **C** ) Staircase deformity on CT scan (red arrow).

**Fig. 15:**
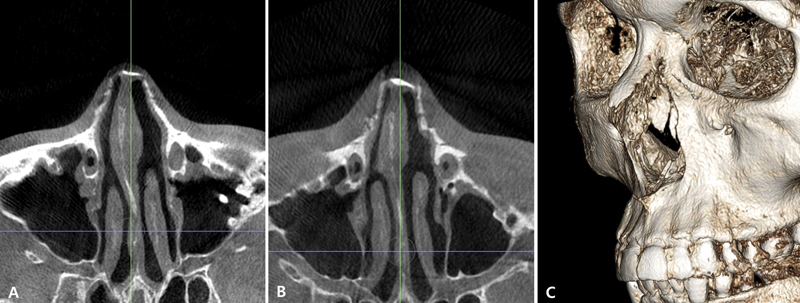
Double-level lateral osteotomy for correction of left-side asymmetric bone. ( **A** ) Preoperative axial view on CT scan. ( **B** ) Postoperative axial view on CT scan. ( **C** ) Postoperative 3D view on CT scan.

The dorsal edges of osteotomized bone may be perceptible, and bony spicules or abnormalities may emerge, which are the downsides of the classical medial osteotomy. This phenomenon is called “rocker deformity” 25 ( Fig. 14A ). In other words, the cephalic margin of the osteotomized bone is angulated outward, while the caudal margin is excessively depressed inward in the rocker deformity. Also, as both the starting point and the progress direction are medial, the degree of pivot mobilization increases, which makes the deformity more severe. On the other hand, when performing lateral osteotomy, the more the medial movement and the higher the fracture level are performed, the more chance of a bony gap and palpable edge of maxillary bone called “staircase deformity” occur ( Fig. 14B , C ). A double-level osteotomy can be applied to correct the “staircase deformity.” In addition, the double-level osteotomy is the preferred effective method when there is an excessive lateral wall convexity with asymmetry since unilateral double-level osteotomy works well to balance asymmetries 26 ( Fig. 15 ).

Various osteotomy methods have been introduced for lateral osteotomy depending on the fracture level. If the osteotomy is performed in the inferolateral area of NMTZ, it will be described as low pattern, while performing the osteotomy superomedial area of NMTZ will be described as high pattern. In the last decade, the authors' method has changed from high-low-high to low-to-low pattern for several reasons. First, by checking the CT scan preoperatively, it is possible to decide the level of osteotomy without damaging the lacrimal passage. Therefore, level of lateral osteotomy gets lower than before. Second, the relationship between the neck of inferior turbinate and pyriform aperture is different in Asian nose: our experience has shown relatively deeper, lower positioned Webster's triangle in Asian patients in CT scan. In such cases, we can prevent the airway obstruction after the osteotomy with combined inferior turbinoplasty. 17

### Technical Tips for Successful Osteotomy

During the medial osteotomy, 3 to 6 mm, curved or straight osteotome is commonly used depending on the surgeon's preference. Recently, a piezoelectric instrument is also used in nasal osteotomies. 27 Electric saw is useful to cut the bone easily, but once making a complete fracture, it is difficult to keep stability, causing bony gap or missing. 28

It is important that the nasal mucosa is not penetrated during osteotomy, and in particular, when an implant is used as it may cause chronic inflammation or contracture. Therefore, surgeon should be careful while performing hump resection or medial osteotomy when implant is used together in Asian nose. Since the overlying dorsal skin of the bony portion where the medial osteotomy starts is very thin, attention should be paid to prevent irregularity or injury of skin. If the medial or paramedian direction is inconsistent with the natural cleavage line of bone, the osteotomy must be performed more cautiously. Regionally, the thickness of the nasal bony vault varies. According to the study on 17 Caucasian cadavers, the bony thickness is increased from caudal (1.93 mm) to cephalic (5.87 mm), while it is also increased from lateral caudad (2.22 mm) to medial cephalic direction (4.41 mm) along the lateral bony wall. 29 All cadavers presented with the transition zone of bony thickness along the bony vault that creates the natural cleavage for the medial osteotomy. Following the natural cleavage plane during medial osteotomy, when combined with lateral osteotomy, 15-degree medial osteotomy produced sufficient narrowing and established a predictable location for smooth greenstick infracture, which was reliable and well-controlled, resulting in a smooth contour of the upper lateral bony vault, while 0-degree osteotomy produced was not reliable as well as unpredictable greenstick fracture and upper lateral bony vault abnormalities by cutting across the natural cleavage plane into thicker bone although the narrowing was adequate. 29 30

An internal continuous osteotomy is performed using a guarded osteotome. The guard was designed to be positioned endonasally and protect the intranasal mucosa, but this location may harm the intranasal mucosa. 31 As a result, guard with osteotomes placed lateral to the nasal bone are routinely employed. Using osteotome with a bigger guard makes it easier for surgeon to palpate to know the level of osteotome, but only at the expense of the soft tissue trauma. The 4-mm low-profile guarded osteotome was shown to be overly damaging, with most patients developing intranasal mucosal tears, whereas the 2.5 to 3 mm guarded low-profile osteotome generated much less damage to the soft tissue. 32 When performing the lateral osteotomy, it is adequate to use osteotomes which are less than 3 mm. A study by Kuran et al showed that the bony thickness throughout the osteotomy line does not exceed 3 mm in many cases. 33 Regardless of lateral wall thickness, the 2-mm osteotome may be utilized effectively for percutaneous osteotomy. 31 Using wide or large guarded osteotome easily causes mucosal tearing that leads to more postoperative bleeding, ecchymosis, and edema. 31 32 33

When performing medial oblique or paramedian oblique osteotomy and lateral osteotomy at the same time, both cephalic end of osteotomy lines should not meet, and it is recommended to leave a gap of at least 2 mm. 23 This small attachment provides the stability and enables to perform the greenstick fracture without causing collapse or sinking of fractured bone. Moreover, when performing medial and lateral osteotomy at the same time, attention should be paid to severe medial or downward displacement of osteotomized bone. If it occurs during operation, a reverse osteotomy with outward fashion, or closed reduction and molding with intranasal packing should be done.

The hammering by assistant during osteotomy procedure plays an important role. All osteotomy tapping with mallet by assistant should be performed very gently and accurately. The transfer of striking force should be carefully coordinated and managed with the assistant. Generally, the learning curve for percutaneous lateral osteotomy is longer than internal. Because small and sharp instrument is used, the lateral osteotomy may be difficult for less experienced surgeon in controlling and obtaining a better result. Due to this difficulty, repeated passing may lead to higher chance of tissue injury, bleeding and malfracture.

### Authors' Method about Perforating Lateral Osteotomy in Practice



**Video 2** Comparison of internal and external approach in lateral osteotomies.


**Video 3** Surgical demonstration of paramedian oblique and percutaneous lateral osteotomy.


Authors prefer percutaneous lateral osteotomy for several reasons. As compared with the intranasal continuous approach, the percutaneous osteotomy results in less bleeding and edema as well as shorter downtime. 2 Furthermore, the percutaneous method preserves the underlying periosteal attachments, resulting in improved overall stability after reposition. 31 Moreover, by conducting the percutaneous lateral osteotomy, less periosteum or mucosa violation was obtained in our previous study. 2 Minimized periosteal elevation may offer the stability as an internal splinting action for safe support of the osteotomized bones. 23 Our cadaver demonstration also revealed that the percutaneous lateral osteotomy provided stronger support for fractured bone than intranasal continuous osteotomy 2 ( Video 2 . Comparison of internal and external approach in lateral osteotomies).

1 Local anesthetic injection—lidocaine with 2% epinephrine
2 To prevent the damage of keystone area, osteotomy is usually performed before septal approach including septal cartilage harvesting, septoplasty, and septal reconstruction.
3 Medial osteotomy should be performed before each lateral osteotomy. Paramedian oblique osteotomy is our preferred technique, and it is generally initiated at the bony cartilaginous junction, 2 to 3 mm lateral from midline. And then, osteotomy is performed in an oblique direction following the natural cleavage line until medial canthal level.
4 The skin incision for lateral osteotomy is placed on the LAL, where the widest aspect of the BW by making an approximately 2 to 3 mm incision along the intended line of lateral osteotomy.
5 The straight osteotome (2 mm) is engaged to periosteum touching the bony surface with one corner of the distal end of the osteotome and osteotomy is initiated in the widest portion of the lateral bony wall in a scratching pattern to make a grooved hatch. The sequence proceeds from distal to proximal and back to distal (back and forth) so that the distal lateral bony pyramid moves inward. It proceeds in the low-to-high or low-to-low direction until the medial canthal level. We prefer to perform a low-low-low pattern as this technique allows us to mobilize a large segment of osteotomized bone minimizing the occurrence of the staircase deformity.
6 During osteotomy, once osteotome is engaged onto the bony surface, try to keep the contact between the osteotome instrument and bone until complete osteotomy. In other words, it is important to not withdraw the osteotome as much possible until osteotomy is complete. This prevents unwanted fracture or irregular osteotomy line.
7 Once the osteotomy line is confirmed after two to three times repeated back and forth scratching, the osteotome is erected more vertically and pushed medially followed by striking to induce the mobilization in a dot pattern. Surgeon should be accustomed to the distinctive fracture sound announcing an imminent fracture.
8 It is possible to obtain more medialization of the bone with transverse osteotomy connecting with lateral osteotomy line at the cephalic end, without performing the medial osteotomy.
9 After osteotomy is finished, a gentle pressure can be given with thumb, index and third fingers to mobilize the osteotomized bone medially followed by molding and check.
10 Once the osteotomized bone is appropriately positioned, dorsal contour and line should be evaluated using palpation test. If the dorsal edge or spicule is palpable, a fine and sharp rasping can be attempted carefully.
11 If there is a risk of airway compromising, we perform preventive turbinoplasty simultaneously in case of high-risk patients.
12 Skin incision is repaired using 7–0 nylon suture to prevent the formation of visible scar.

External lateral osteotomies are authors preferred technique as follows: ( Video 3 . Surgical demonstration of paramedian oblique and percutaneous lateral osteotomy)

## Conclusion

Nasal osteotomy is a crucial and challenging aspect of rhinoplasty, particularly in Asian populations. This study intended to improve the understanding of nasal osteotomy with consideration on surface aesthetics and nasal anatomy. The surface aesthetics of the nose, including the dorsal and lateral aesthetic lines, dorsal and basal widths, play a significant role in determining the appropriate osteotomy techniques. Since Asian noses have distinct anatomical differences compared with Caucasian ones, making the procedure more complex and requiring careful attention. Surgeons must comprehend the relationship between surface aesthetics and osteotomy techniques to achieve consistent and reproducible results. Overall, a comprehensive understanding of surface aesthetics, nasal anatomy, and the relationship between osteotomy techniques and their effects on surface aesthetics is crucial for successful nasal osteotomies in Asian rhinoplasty.
